# Antiviral Nanomaterials for Designing Mixed Matrix Membranes

**DOI:** 10.3390/membranes11070458

**Published:** 2021-06-22

**Authors:** Abayomi Babatunde Alayande, Yesol Kang, Jaewon Jang, Hobin Jee, Yong-Gu Lee, In S. Kim, Euntae Yang

**Affiliations:** 1 School of Civil, Environmental and Architectural Engineering, Korea University, Seoul 02841, Korea; abayomi@korea.ac.kr; 2 School of Earth Sciences and Environmental Engineering, Gwangju Institute of Science and Technology (GIST), Gwangju 61005, Korea; yesol7964@gist.ac.kr (Y.K.); sejane13@gmail.com (J.J.); iskim@gist.ac.kr (I.S.K.); 3 Department of Marine Environmental Engineering, Gyeongsang National University, Tongyeong-si 53064, Korea; hbj99@gnu.ac.kr; 4 Department of Environmental Engineering, College of Engineering, Kangwon National University, Chuncheon-si 24341, Korea; yonggulee79@gmail.com

**Keywords:** antiviral, virucidal, mixed matrix membrane, nanomaterial

## Abstract

Membranes are helpful tools to prevent airborne and waterborne pathogenic microorganisms, including viruses and bacteria. A membrane filter can physically separate pathogens from air or water. Moreover, incorporating antiviral and antibacterial nanoparticles into the matrix of membrane filters can render composite structures capable of killing pathogenic viruses and bacteria. Such membranes incorporated with antiviral and antibacterial nanoparticles have a great potential for being applied in various application scenarios. Therefore, in this perspective article, we attempt to explore the fundamental mechanisms and recent progress of designing antiviral membrane filters, challenges to be addressed, and outlook.

## 1. Introduction

Pathogenic viruses have been considered a significant threat to human civilization. And, due to the COVID-19 pandemic, it has become a more significantly visible concern. The attention towards antiviral technologies has significantly soared worldwide to prevent the ravages of viruses [1,2]. Among the antiviral technologies, the development of vaccines and antiviral drugs is the most sure-fire strategy to contain epidemic viral diseases [3]. However, to invent new antiviral vaccines and medicines, enormous efforts and a substantial amount of time are required. Thus, in a situation without effective vaccines, the prevention of contact with viruses through physically filtering (i.e., membrane filtration) and chemically destroying viruses (i.e., disinfection) is the most critical approach to avert viral transmission [4,5,6].

Among the two main strategies, despite excellent virus-killing capability, chemical disinfection has several recognized drawbacks, such as generating harmful disinfection byproduct, safety concerns for human health and environmental impact, and the existence of viruses resistive to disinfections [7]. On the other hand, membrane filtration technologies can selectively reject all kinds of viruses from water and air using a physical barrier and selective transport without creating harmful byproducts and safety concerns for human health. In addition, membrane filtration technologies are simple, reliable, efficient, and readily applicable compared to other technologies [8]. Thanks to those advantages, membrane filtration technologies have widely been applied to block the inflow of both airborne and waterborne viruses. In addition, they have been broadening the domain of their application in many fields [9].

### 1.1. Virus Removal Mechanisms of Membrane Filters

Membrane filters can eliminate viruses from air and water based on various mechanisms, such as size exclusion, hydrophobic interactions, electrostatic interactions, inertial impaction, and diffusional interception [10,11]. Each mechanism’s contribution can be varied depending on several factors (e.g., environment, membrane properties, and operational conditions). Here, we briefly explain the mechanisms of membrane filtration based on the environment where membranes are applied, that is water and air filtration.

**Figure 1:**
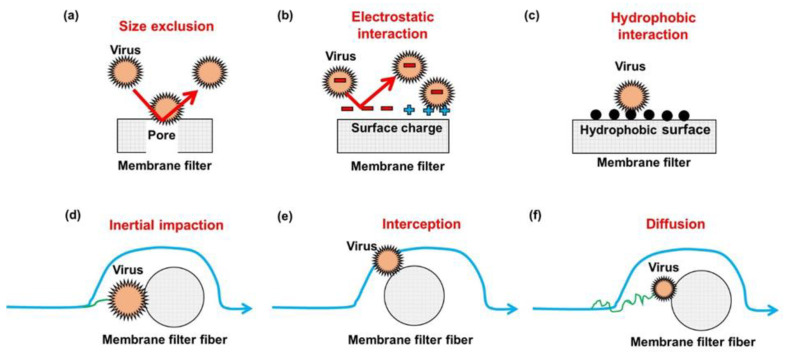
A schematic diagram of the mechanisms by which membrane filters remove airborne and waterborne viruses.

For water purification membranes, the predominant mechanism is generally size exclusion. Size exclusion can be intuitively explained by that larger-sized viruses are rejected by smaller-sized pores of membrane filters (Figure 1a). The size of viruses usually ranges from 20 nm to 200 nm based on the types of viruses [12]. Based on pore size, membrane filters for virus removal from liquids generally belong to the range between microfiltration (MF) and ultrafiltration (UF), which are classified into porous membranes [13]. Furthermore, non-porous membranes, such as nanofiltration (NF) and reverse osmosis (RO), can effectively remove viruses based on size exclusion [14]. Besides size exclusion, as illustrated in Figure 1b,c, viruses can be removed by the adsorption predominantly governed by hydrophobic and electrostatic interactions between viruses and membrane surfaces. In addition, if viruses and the membrane surface have the same charge, electrostatic repulsion contributes to virus removal [11].

Similarly, a size exclusion-based sieving mechanism is involved in most air filter designs. For air filtration, size exclusion occurs when the size of the particles is larger than the pore of the filters. The particulate sieving is entirely determined by the size of the target viruses, the diameter of the membrane pore, and media density. The size of infectious aerosols is less than 5 μm, and that of virus-laden respiratory droplets that are inhalable by humans is less than 20 μm [15]. In addition, electrostatic and hydrophobic interaction works to retain the viral contaminants during air filtration [16,17]. However, unlike water filtration membranes, other mechanisms such as inertial impaction, interception, and diffusion importantly serve to remove contaminants in air filtration membranes [18]. Inertial impaction transpires when large-sized particulates (for example, atmospheric aerosol particles) cannot quickly adapt to the rapid changes in air streamline near a fiber of filter, as shown in Figure 1d. Interception is involved in capturing certain-sized particles that move along with air streamlines flowing sufficiently close to a filter fiber. The certain-sized particles physically contact a filter fiber, and they become attached to the filter fiber (Figure 1e). The diffusion mechanism predominantly works to retain very small-sized particles with a diameter of less than 0.1 μm. Owing to the interaction between the small-sized particles and Brownian-motioning gas molecules, the small-sized particles are randomly motioned and can bump into a fiber of filter (Figure 1f).

### 1.2. Addition of Virucidal Capability to Membrane Filters

Although the extraordinary efficiency of virus removal of filter-based physical separation is important, the rejected pathogenic viruses need to be safely disposed of. Particularly in water purification, if the filtered pathogenic viruses in retentate are not appropriately treated, these can remain a continuous risk for infection. This risk could be reduced by adding virucidal function to membrane filters. In addition, the antiviral ability can make membrane filters more efficient for treating pathogens by synergizing with physical separation. 

Antiviral membrane filters have been developed by incorporating virucidal nanomaterials into membrane matrices, generally referred to as mixed-matrix membranes (MMMs) [19,20,21,22]. Various biocidal nanomaterials (for example, silver nanoparticles and copper nanoparticles) have been used to fabricate antiviral MMMs for air and water purification [19,20,21,22]. To date, based on our survey using Web of Science, research articles focusing on antiviral MMMs have been published in the last several years. Among these articles, there are only a few review articles covering antiviral MMMs, despite soaring interest in research on antiviral materials [14,23,24,25,26,27,28,29,30,31]. Moreover, those review articles simply address antiviral MMMs only as a small part of their evaluation focused on membrane processes aimed at virus removal [14,27,29], electrospun nanofibers [24,25], disinfection technologies [28,30,31], or face mask filters [23,24,26]. Therefore, our present work intensively focuses on the development, application, and future perspectives of antiviral MMMs for air and water purification.

### 1.3. Scope of This Review

In this work, in order to lay the groundwork for discussion, the general background behind research on membrane filtration technologies for virus removal is introduced. Then, to offer insights to those looking for the feasibility of virucidal nanomaterial-based MMMs, a concise review of currently available studies on antivirus nanomaterials and virucidal membrane filters for air and water purification are examined. Finally, the current and potential applications of antiviral MMMs are discussed. This work also provides researchers new research directions for designing antiviral MMMs.

## 2. Antiviral Nanomaterials

Nanomaterials refer to materials ranging in size of 1−100 nm in at least one dimension and exhibit completely different physicochemical properties from their bulk. The potential application of nanomaterials to fight pathogenic and unwanted microbes has been extensively documented [32,33]. Nanomaterials can be categorized into organic and inorganic nanomaterials. Organic nanomaterials such as polymeric nanomaterials are mainly used as drug delivery agents due to their easy uptake by cells. Examples of organic nanomaterials include liposomes, micelles, dendrimers, solid lipid nanoparticles, etc. Organic antiviral nanomaterials may also enhance the efficacy of antiviral drugs [34]. Inorganic nanomaterials, on the other hand, have been extensively used to eradicate viruses in numerous applications such as construction, medicine, separation, and textiles. In the field of membrane-based separation, antifouling, self-cleaning, and overall membrane performance can be significantly improved using nanomaterials. Examples of antiviral nanomaterials include but are not limited to silver nanomaterials, gold nanomaterials, copper nanomaterials, titanium oxide nanomaterials, zinc oxide nanomaterials, and carbon-based nanomaterials (Table 1).

### 2.1. Silver Nanomaterials (AgNMs)

Silver (Ag) is a soft, whitish, shiny metal with characteristic high thermal and electrical conductivity. The physicochemical properties of AgNMs play a significant role in their virucidal ability. A high specific area and small size enhance viral interaction, rate of Ag ion generation and release, and reactive oxygen species (ROS) production. For example, Mori et al. [57] investigated the antiviral properties of AgNMs embedded in polymer matrix against the H1N1 influenza A virus. The results of the study showed that the polymer (chitosan) showed no antiviral activity; however, the incorporation of AgNMs in the matrix of the polymer enhanced the antiviral efficacy. Although the actual antiviral mechanism was not established in their study, improved antiviral activity of the matrix was attributed to AgNMs concentration and size. This demonstrated that AgNMs concentration and average size are important factors that influence AgNMs’ antiviral properties. In addition, the morphology and oxidation state of AgNMs affect their virucidal ability [58]. Some of the modes of AgNMs’ antiviral action include the release of Ag^+^ ions causing membrane and genome damage, viral protein blocking, and the suppression of reverse transcription [59]. Occasionally, the antiviral mechanism of Ag may depend on the type of virus. AgNMs have been explored in membrane-based water treatment for the removal and destruction of viruses. Zodrow et al. [36] incorporated AgNMs into polysulfone membranes used in water treatment to enhance their antiviral properties. The Ag-incorporated polysulfone membrane was tested against MS2 bacteriophage. The complete viral removal efficiency was observed for the AgNM-incorporated membrane, while polysulfone membrane alone could not eliminate the virus from the membrane effluent. The increased viral treatment of the membrane was attributed to Ag ion release. In a related study, Gusseme et al. [60] fabricated a silver-polyvinylidene fluoride MMM for UZ1 bacteriophage deactivation. The results show that the gradual release of Ag ions from the membrane was effective in deactivating viruses and enhancing a long-lasting antiviral property. Ju et al. [61] synthesized a nanofibrous air filter membrane for high-efficient particulate matter (PM) removal and antiviral and antibacterial properties. The membrane was fabricated by anchoring AgNMs to the polyamide through hydrogen bonding. The antiviral property of the air filter was tested against porcine Deltacoronavirus. A decreased virus titer was reported as virus contact time with the membrane increased. This was ascribed to the antiviral property of AgNMs. Apart from AgNMs, other derivatives of Ag such as Ag_2_S nanocluster (NCs) have also been reported to possess excellent antiviral properties [39].

### 2.2. Gold Nanomaterials (AuNMs)

The most available literature of AuNMs is focused on its use as a drug carrier. However, there is evidence of the virucidal efficacy of AuNMs on viruses. Gold (Au) can adhere to biological materials to enact cell deactivation tendencies. The blocking of binding sites of viruses by AuNMs prevents the attachment of the virus to its host, thus preventing viral proliferation. For example, Rafiei et al. [62] evaluated the antiviral activity of AuNMs against the foot-and-mouth disease virus (FMDV). The authors reported around four-fold FMDV titer reduction compared to the control test. The actual mechanisms of antiviral action were not clearly stated in the study; nevertheless, a better viral inhibition was observed at the later stages of viral replication compared to the early stage of infection. Similar to AgNMs, AuNMs’ antiviral potency can be enhanced by controlling their average size and morphology [41,63].

### 2.3. Copper Nanomaterials (CuNMs)

The superior contact killing of copper-based NMs makes them a promising candidate for cleaning and disinfection, construction, medicine, water treatment, and textile applications [64,65]. The use of Cu-based films for viral deactivation has increased with the COVID-19 pandemic, where publicly touched surfaces are covered with Cu-based films to prevent the spread of COVID-19. The success of this material is due to CuNMs’ capacity to disrupt viral integrity and degrade its genome on contact. Cu-based derivatives can exhibit different antiviral activity depending on the valency of the CuNMs [66]. The physical state, such as size and Cu ion release, can affect the efficacy of CuNMs. Cu ion release from CuNMs has been reported to catalyze ROS generation and cell membrane depolarization [42]. For instance, human influenza A virus (H1N1) and avian influenza virus (H9N2) was effectively deactivated without compromise in the integrity of a CuONMs incorporated N95 face mask [67]. In another study, cuprous oxide nanomaterials (Cu_2_ONMs) were tested against hepatitis C virus (HCV), and 90% virions reduction was observed [43].

### 2.4. Zinc Oxide Nanomaterials

ZnONMs have attracted attention for their applications in various areas concerning biomedicine, energy storage, electronics, optics, and physical chemistry [68,69]. However, the antiviral activity of ZnONMs has received relatively lesser attention compared to other nanomaterials. However, the antiviral property of ZnONMs is documented in the literature [44]. ZnONMs are semiconductor photosensitizers that can produce ROS through the excitation with light owing to their optical and catalytic properties [68]. ZnONMs are a potent antiviral agent because they possess other modes of antiviral action, such as viral inactivation via non-ROS pathways and Zn^2+^ ion release, which causes polymerase function inhibition [45,46]. These properties of ZnONMs make them a suitable material for both self-sanitizing and antiviral applications.

### 2.5. Titanium Oxide Nanomaterials (TiO_2_NMs)

TiO_2_ is a widely used semiconductor for antimicrobial application due to its low cost, low toxicity to humans, and good stability [70]. The antiviral property of TiO_2_ is majorly dependent on its photodynamic inhibition (PDI) (described in Section 3). TiO_2_ is one of the most effective PDI agents because of its ability to donate the electron generated at the conductive band to oxygen and simultaneously use the hole generated at the valence band to produce ROS. The antiviral activity of TiO_2_NMs is then performed by ROS, which may destroy components of viruses such as membrane, DNA/RNA, and proteins. The effectiveness of TiO_2_NMs depends on their size, surface-to-volume ratio, morphology, stability, bandgap, reduced charge carrier recombination, and absorption at a wide light spectrum. The generated ROS may either react with the cell wall or cell membrane to cause their disruption. TiO_2_NMs can also demonstrate antiviral activity by damaging the lipids in a viral envelope by the action of G-sol [47,48].

### 2.6. Carbon-Based Nanomaterials

Carbon-based NMs include fullerene, carbon quantum dots, single-walled and multi-walled carbon nanotubes (SWCNT and MWCNT), graphene, graphene oxide (GO), and reduced graphene oxide (rGO). These carbon-based NMs have shown antiviral properties in numerous applications [71]. The carbon atoms link with each other in different ways and with different binding energies to exhibit different dimensions such as 0D, 1D, and 2D. The antiviral properties of carbon-based NMs can differ based on the type of carbon-based NMs and their dimension. For instance, fullerenes can inhibit viral maturation and replication [49], whilst quantum dots can disrupt viral replication by inactivating the interferon response for both RNA and DNA viruses and the production of ROS [72,73]. The antiviral ability of GO and rGO has been connected to their unique structure (membrane piercing) and highly negative surface charge [52]. Similarly, CNTs exhibit viral mechanical damage by puncturing and straining the virions [74]. Furthermore, the electrical, thermal, mechanical, and chemical properties of carbon-based NMs can be harnessed to improve the antiviral properties of these materials.

### 2.7. Silica Nanomaterials

Silica is a nontoxic and inert material that can be easily engineered to impact additional surface properties [75]. The modification of silica’s surface properties, such as hydrophobicity and charge, enhances the affinity of these nanomaterials with viruses. Silva et al. [55] synthesized SiNMs with different function groups grafted on the surface with consistent shape and size. The antiviral property of SiNMs was enhanced when the grafted function group was able to easily interact with the viral envelope.

### 2.8. Tin Oxide Nanomaterials (SnO_2_NMs)

SnO_2_ is a good semiconductor, which has a large bandgap. The physicochemical properties, most especially grain size and shape, of SnO_2_NMs make them promising antiviral materials. The small size of SnO_2_NMs increases its surface area and subsequently increases activities. The antiviral properties of SnO_2_NMs were investigated by Trigilio et al. [56]. In their study, the negative charge of SnO_2_NMs was used to bind and trap HSV−1 before its entry into the host cell. The authors discovered that the binding of SnO_2_NMs with the viral particle inhibits viral entry, replication, and prevents cell-to-cell fusion [56]. 

## 3. Mechanisms of the Antiviral Activity of Nanomaterials

**Figure 2:**
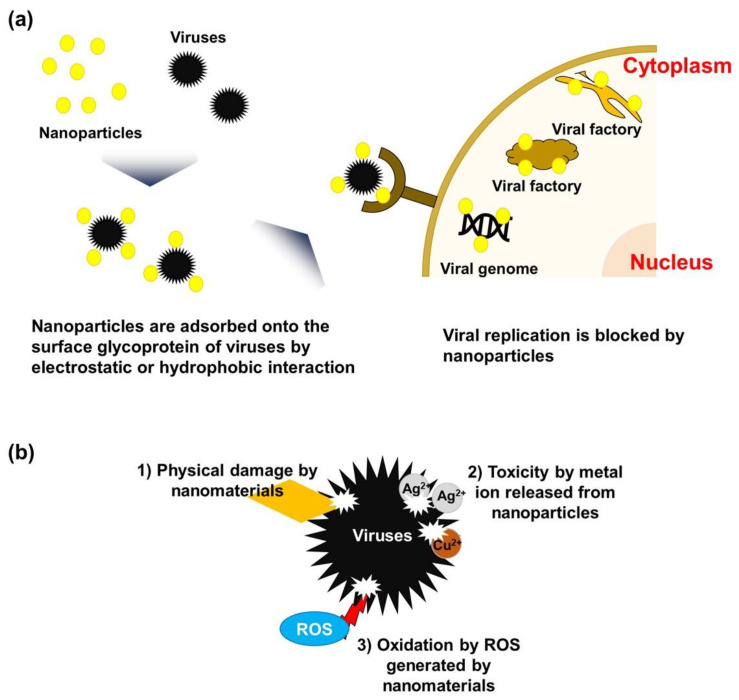
Scheme illustrating working principle of virucidal nanomaterial for inactivation of viruses. (**a**) A mechanism of blocking the replication of virus by nanoparticles, and (**b**) principles for virus killing by chemical and physical attacks by nanoparticles.

Nanomaterials can possess desirable chemical, mechanical, electrical, and optical properties, depending on their size, surface area, atomic composition, surface morphology, and surface energy [2]. Thanks to these unique properties, nanomaterials can effectively inactivate or even destroy viruses. Many previous studies have investigated the virostatic and virucidal mechanisms of various nanomaterials [4,34,76]. Figure 2 illustrates the verified or expected virostatic and virucidal mechanisms of nanomaterials. Generally, the virostatic and virucidal activities of nanomaterials can be generated through direct physical contact, chemical oxidation by reactive oxygen species (ROS), metal ionic species release, and photothermal effects [4,34,76].

Firstly, nanoparticles such as silver and copper nanoparticles can inactivate viruses by disrupting the invasion of viruses into cell receptors through interaction with surface proteins or envelopes of viruses [77,78], as shown in Figure 2a. Owing to the hydrophobic and negatively charged surface of viruses, hydrophobic and electrostatic interaction is predominantly contributed to adsorption between viruses and other solid surfaces [2]. Thus, to further enhance the interaction with targeted viruses, the surface chemistry of nanoparticles has been manipulated by surface coating and functionalization [79,80].

Nanostructured sharp edges of nanomaterials can also directly damage the viruses when nanomaterials come into contact with viruses (Figure 2b). It was found that the viral envelope and spikes were devastated when the sharp edges of GO and rGO nanosheets came directly in contact with the viruses [52,81]. In addition, metal ions released from metal nanoparticles can inactivate viruses [82] (Figure 2b). Although our understanding of the mechanisms of antiviral action by metal ions is still in its early stages, the viricidal properties of these metal ions are believed to depend on the type of virus. For example, copper and silver ions can damage the viral genome and/or disrupt the viral membrane [82], while zinc ions can inhibit RNA polymerase [83].

Finally, viruses can be inactivated by the PDI property of nanomaterials, especially by the semiconducting nanomaterials. PDI refers to the inactivation of infectious agents by the production of radicals by photosensitizers (e.g., semiconductors). The interaction of these nanomaterials with light generates ROS such as hydroxyl radicals and superoxide radicals [84]. When semiconducting nanomaterials are excited by light, an electron-hole pair is produced. The conduction band electron produced can be accepted by oxygen to generate superoxide radicals, which can further be converted to hydroperoxide radicals, hydrogen peroxide, and eventually, hydroxyl radicals, while the valence band hole produced can split water to generate hydroxyl radicals (free and surface-bound). This ROS can induce damage to viral membranes, proteins, and nucleic acids (RNA/DNA) [85] (Figure 2b). The PDI property of nanomaterials could be more beneficial during water treatment because both parts of the electron-hole pair can be utilized for a radical generation while only the available oxygen can be used as an electron acceptor to produce radicals during air filtration.

## 4. Development of Antiviral MMMs

By leveraging on the physical filtration and antimicrobial properties of nanomaterials, the incorporation of antiviral nanomaterials into the matrix of a polymer would not only help overcome the trade-off between permeability and selectivity of polymeric membranes but also confer antifouling and contact microbial killing properties. Nevertheless, before the introduction of any type of antiviral nanomaterial into the matrix of polymer, it is important to examine the compatibility of the nanomaterials with the polymer. This is because the compatibility between nanomaterials and polymer plays a significant role in the performance of the membrane. In addition, lack of compatibility could cause the leaching of nanomaterials out of the polymer matrix and thus compromise membrane integrity. The compatibility of nanomaterials can be determined based on their chemical structures, surface chemistry, and size distribution [86].

**Figure 3:**
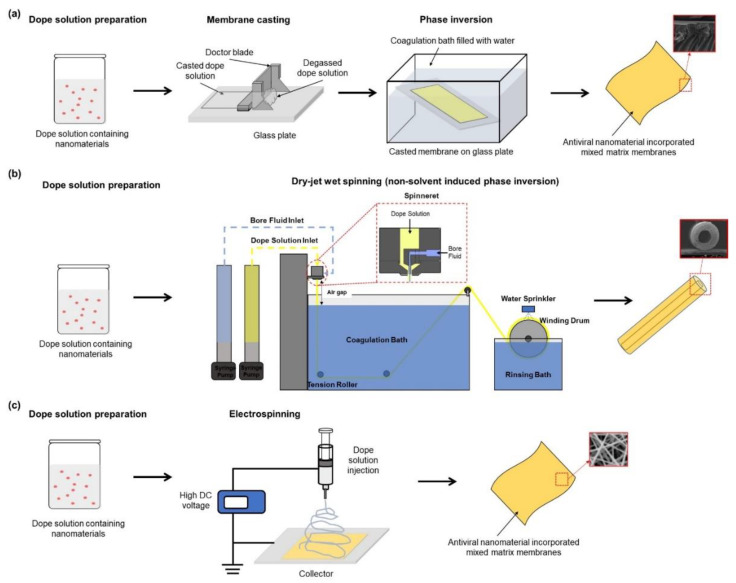
Fabrication methods of antiviral MMMs: (**a**) Phase inversion, (**b**) non-solvent induced phase separation by dry-jet wet spinning, and (**c**) electrospinning.

The synthesis and fabrication of conventional polymeric membranes have been developed to prepare MMMs. Therefore, antiviral MMM can be fabricated on a large scale by simply adopting current strategies for the fabrication of polymeric membranes. An essential initial step for the preparation of antiviral MMM is dope solution preparation. The dope solution can be prepared in three ways [86]: (1) Dispersing the nanomaterials in solvents and then adding the polymers, (2) dissolution of polymers in the solvents before adding the nanomaterials, or (3) dispersing the nanomaterials and dissolving the polymers in separate solvents before mixing. To avoid aggregation of nanomaterials, (1) and (3) above are best suited for fabricating antiviral membranes. When the polymer is eventually added, the tendency for nanomaterials aggregation is low compared to when the polymer is initially dissolved in the solvent. After achieving well-dispersed nanomaterials in the dope solution, the dope solution should be allowed to degas to remove all the bubbles in the solution. The presence of bubbles would result in defeat and create holes on the membrane if not removed completely. After that, the antiviral MMMs can be mainly fabricated via phase inversion or electrospinning (Figure 3).

Phase inversion has been employed for the fabrication of antiviral MMM [36,60]. Phase inversion is the process of transforming a dope solution in a liquid phase into a solid phase by casting/spinning it into a flat sheet/hollow structure. The antiviral MMMs can be prepared in a sequence of two steps. First, the solvent is allowed to partially evaporate (evaporation), and second, the cast/spun dope solution is submerged in a non-solvent coagulation bath to solidify the cast composite material. The evaporation step allows the development of a thin skin layer at the topmost layer of the cast/spun composite because of solvent evaporation. This layer is mostly responsible for membrane flux, selectivity, and antimicrobial properties. The solvent-nonsolvent exchange process that takes place in the coagulation bath allows the non-solvent to diffuse into the polymer while the solvent diffuses out through the layer formed during the evaporation step. The thermodynamic instability that occurs during this step determines the type of porous structure that would be formed beneath the dense thin layer. Moreover, the time allowed for solvent-nonsolvent exchange can determine the porosity of the membrane. Even though the phase inversion method of fabricating MMMs is simple, achieving the best membrane performance is often difficult. Several strategies have been adopted to enhance the performance of MMMs by annealing post-treatment, polymer composition adjustment (e.g., polymer type, polymer concentration, additive inclusion), and casting/spinning conditions (e.g., composition, temperature, humidity) [87]. After casting the MMMs, a residual solvent can be removed by drying post-treated in an oven at a specific temperature allowable by the polymer glass transition temperature.

For hollow fiber-type MMMs, a phase inversion-based dry-jet wet spinning technique is employed. Since Dow Chemical Company developed a manufacturing system of hollow fiber membrane in the 1960s, this technique has been intensively used in various fields. Hollow fiber membranes have a small outer diameter; thus, packing hollow fiber bundles in the module has the effect of greatly improving the specific surface area of the membrane. In general, the hollow fibers can be fabricated by non-solvent induced phase separation (NIPS) and thermally induced phase separation (TIPS) methods. In NIPS, especially, the hollow fibers are formed via solidification by discharging the spinning solution dissolved in a dope solvent with a polymer resin through the spinneret and contacting the discharged bore solution. In addition, it is possible to fabricate MMM hollow fibers with the desired composition and properties by using the appropriate composite solution in the NIPS process [88,89,90]. 

Electrospinning is another simple and versatile strategy to fabricate an antimicrobial mixed matrix nonwoven mesh of micro- or nanofiber [91]. The dope solution is loaded in a syringe and needle, and a high electric voltage is applied to the dope solution and a collector. The application of high voltage forces the dope solution through the needle to form a jet, which gets dried and collected on the collector. Several parameters, such as conductivity, viscosity, operating voltage, surface tension, pressure, temperature, and flow rate, can influence the electrospinning process. However, the dope solution viscosity, surface tension, and conductivity are believed to be critical in electrospinning [92]. For example, viscosity determines chain entanglement within the solution [93]. The micro or nanofibers can then be dried, hot pressed, or post-treated under high heat. Large-scale production of electrospun MMM can be fabricated by using multi-jet and blowing-assisted electrospinning technologies [94,95]. 

Except for those fabrication techniques, other techniques are possibly employed to fabricate the MMM, such as layer-by-layer (LBL) coating and hydrogel or aerogel fabrication. When fabricating the antiviral MMM with these processes, the membranes can be made using composite materials which have antiviral properties. These types of MMMs are intensively used in water treatment, desalination, oil/water separation, etc.; they showed excellent purification or separation performances and antifouling property. A detailed description of each fabrication method is presented below.

The LBL method has been used in many studies since it was first reported in the 1990s, and it is a facile method that can produce the MMM layer on the support at a relatively low cost [96,97]. The LBL method, a type of molecular self-assembly technology, uses the electrostatic attraction of the materials with positive/negative charges as a driving force to coat the substrate. The thickness of the active layer can be effectively controlled by stacking materials one by one on an atomic scale. Usually, the thickness of the layer increases linearly in proportion to the number of LBL coating processes, but when coating material with spontaneous diffusion properties, the thickness change is non-linear or exponential characteristics. The morphology of the membrane can also be improved by controlling the state of the precursor solution or the process conditions. In addition, the LBL process is applicable in various situations and can be implemented with various protocols such as soak/wash cycles, spin, and spray treatments to control the properties of the final product. In order to improve the long-term operation stability of MMM fabricated by the LBL method, the membrane surface can be strengthened by inducing a chemical crosslinking process. Recently, various organic/inorganic MMMs using the LBL method have been reported in water treatment fields [98,99,100,101,102].

In addition, a structure or performance that is difficult to demonstrate by conventional membrane fabrication methods can be implemented with hydrogel or aerogel membranes. This novel membrane structure can be fabricated using many combinations of nanomaterials such as graphene, polymer, metal, metal-oxide, and metal-organic framework, and can be applied in various situations according to the application conditions because there are multiple methods such as polymerization, ultraviolet curing, sol-gel reaction, and hydrothermal reaction to form a hydrogel or aerogel [103,104,105,106,107,108,109,110,111,112].

## 5. Current Application of Antiviral MMMs

Various MMMs having antiviral or bacterial properties are being studied, and many attempts have been made to apply MMM for the purpose of separating/removing/purifying viruses or bacteria in a field of air and water applications. In the case of air treatment, nanoparticles such as zinc oxide (ZnO), silver (Ag), and silica were embedded in the polymer matrix to enhance the antifouling and antibacterial of the air filtration membrane. There also are several cases of using the MMM for water applications: Preventing attachment of foulant at the membrane in desalination or water treatment (antifouling), removing the viruses or bacteria in water (sanitation), and obtaining the pure viruses for a medical purpose, for example (Table 2).

### 5.1. Air

#### Antifouling and Antibacterial in Air Purification 

Membranes for air filtration can be used in two ways: gas separation and fine particles rejection. During air filtration, airborne microorganisms are accumulated on the membrane surface. It eventually deteriorates filtration performance and even can be a secondary bioaerosol pollution source [125]. Therefore, it is important to improve the antibacterial and antifouling activities of the air filtration membrane. Recently, researchers applied antimicrobial additives by incorporating them into a polymeric membrane matrix to fabricate antibacterial membranes. 

Sheida et al. [113] used zinc oxide nanoparticle (ZnO NP) as an additive to achieve antibacterial and photocatalytic properties of the membrane, and it was embedded into the aromatic polyimides (PIs) matrix. Photocatalytic production of hydrogen peroxide (H_2_O_2_), which can degrade the organic pollutants and sterilize the microorganisms, occurred, since ZnO NP has a relatively large bandgap (3.37 eV). Furthermore, it has various characteristics such as large surface area, biocompatibility, intense UV absorption, and high thermal and chemical stability. A bacterial test with the agar disk diffusion method was performed by using common and virulent pathogen agents. The pure PI membrane did not show any inhibition zone for the strains; however, ZnO NP-embedded membranes showed a significantly increased inhibition zone, and it was enhanced when the amount of NP was increased. 

Seong-Min et al. [114] also applied ZnO NP due to its photocatalytic property, which can decompose microorganisms. Furthermore, to grant bactericidal property, silver nanoparticles (Ag NP) were used together. Polyacrylonitrile (PAN)-based membranes were fabricated by an electrospinning technique, but to avoid agglomeration and strengthen the potential of each nanoparticle, side by side nanofibers (SBS NFs) was introduced. It simultaneously ejected the ZnO NP/PAN solution and Ag NP/PAN solution; therefore, it formed a coupled fiber that had photocatalytic properties from ZnO NP and antibacterial properties from Ag NP at the same time. In order to compare the SBS NFs and single nanofibers (SNFs), which contained both ZnO and Ag, randomly, the antibacterial halo-test was performed. *S. aureus* and *K. pneumoniae* were cultured on the LB culture plate, respectively. The inhibition zones of SBS NFs were 32 and 33 mm for *S. aureus* and *K. pneumoniae*, respectively; however, that of SNFs showed only 19 and 21 mm. Through these results, it can be confirmed that SBS NFs had higher antibacterial activity owing to its special parallel nanofiber. Its unique structure helped Ag NP release Ag ions easily and fast without any interruption from agglomerated ZnO particles. 

Saz et al. [115] studied the antifouling and antibacterial properties of silver (Ag)/zinc oxide (ZnO) incorporated graphene oxide (GO) nanosheets (MGO) which were modified by quarternary cetyltrimethylammonium bromide (CTAB), and it was combined in poly (methyl methacrylate) (PMMA)/polyethylene glycol (PEG) membrane. Herein, *E. coli* was used as a model microorganism for testing the antibacterial experiments, and bovine serum albumin (BSA) protein was adsorbed on the membrane surface to carry out the antifouling tests. The antibacterial activity of CTAB@MGO-PMMA/PEG membrane against *E. coli* DH5α strain was measured by agar plate experiment. The pure PMMA/PEG membrane and nanoparticle embedded membranes were compared by the bacterial growth changes depending on the time (0–10 h in an incubator) and inhibition zone changes. CTAB@MGO-PMMA/PEG membrane showed a dramatic reduction of bacterial growth compared to the pristine PMMA/PEG membrane. In the agar plate test, CTAB@MGO embedded membrane had the smallest antibacterial zone than the pristine membrane. The antifouling test also showed similar results to the antibacterial test, as the BSA adsorption value of CTAB@MGO was lowest compared to other membranes. It is because both PEG and CTAB@MGO have hydrophilic moieties, which can weaken the interactions between BSA molecules and membrane surface, resulting in little protein adsorption.

Hashem et al. [116] demonstrated the ultrasonic irradiation applied bio-nano composite membrane, which was composed of polyester (PE) and cellulose/silica nanofillers. Herein, *B. cereus* and *E. coli* were used as target pathogenic bacteria for a disc diffusion assay test. The number of viable colonies of PE/cellulose/silica membrane was decreased compared to the pristine PE membrane. Metal oxide such as silica was an antimicrobial material. Between the negatively charged microorganism and the positively charged metal was an electromagnetic attraction; thus, it oxidized the microorganism and eventually led to death.

Shu et al. [117] prepared polyethersulfone (PES)/polyaniline (PANI) nanocomposite membranes that were fabricated by a phase inversion with well-dispersed PANI nanorods in the casting solution. The PANI nanorod was applied since it can control the surface hydrophilicity and pore size of the membrane. In order to verify the effect of PANI nanorods on the antifouling activity, each membrane filtered 200 mg L^−1^ BSA aqueous solution at 0.2 MPa. The flux recovery rate (FRR) of pure PES membrane was only 48%, but that of PES/PANI membrane was above 60%. Therefore, PANI nanorods were helpful additives to enhance the antifouling property of the membrane since they increased the hydrophilicity of the membrane surface. 

For bioaerosol removal, antimicrobial air filters are commonly used since it is easily and conveniently can be applied to the conventional air-conditioning system. Jeongan et al. [118] introduced the herbal extract incorporated (HEI) nanofiber for fabricating an antimicrobial air filter. *Sophora flavescens* (*S. flavescens*) was used since it has strong antibacterial characteristics against pathogens. Herein, *S. epidermidis* was chosen as a model microorganism for an antimicrobial test. The optimum concentration (6.11%) of *S. flavescens* contained membrane had the highest bacterial inactivation rate (~99.98%). It is because the extracted *S. flavescens* has two major antibacterial chemical compounds, which are sophoraflavanone G (antibacterial) and kurarinone (antioxidant). Therefore, herbal extract/polymer combined nanofiber with enhanced antibacterial activity is a promising candidate for removing bioaerosol.

### 5.2. Water 

#### 5.2.1. Water Sanitation

To improve hygiene by removing bacteria or viruses from water or solution, MMM is used in addition to antifouling purposes. The antibacterial or antivirus properties of MMM eventually result in the antifouling effect mentioned above, but in the case of sanitation, the main concern is not whether fouling is formed on the membrane. The role of MMM in sanitation is to eliminate bacteria or viruses. The sanitation effect of MMM has been reported from quite a variety of research cases. Wang et al. reported that 99.9999% of *E. coli* was removed by a coating of the GO-*graft*-quaternized chitosan composite on the electrode of the water purification system based on capacitive deionization [126]. Jiang et al. fabricated the composite membrane of TiO_2_-Ag-crumpled GO and confirmed the sanitation effect in the ultrafiltration process [119]. As is known, the sharp edges of GO nanosheets or Ag ions can inactivate or kill bacteria. Zhang et al. produced a composite nanofiber membrane by electrospray and electrospinning of a TiO_2_ nanoparticle and Nylon solution, respectively [120]. When this membrane is exposed to UV light in contact with *E. coli*, photokilling occurs due to the photoactive property of TiO_2_ nanoparticles [127]. Cado et al. prepared a bovine cateslytin-cysteine functionalized hyaluronic acid/chitosan composite film and confirmed that it decreased bacteria activity and inhibited proliferation when exposed to *Staphylococcus aureus* bacteria and *Candida albicans* yeasts [121]. The authors predicted that this material could be used as a coating material to prevent infection in medical tools such as catheters and tracheal tubes. In addition, sanitation effects using various types of MMM have been reported, and these MMM are expected to be applied to water treatment or domestic sewage treatment to enhance sanitation performance.

#### 5.2.2. Virus Purification

MMM can be useful even when obtaining high-purity viruses, wherein impurities or byproducts are excluded for use in vaccine manufacturing or biotechnological/medical research. This virus purification is also a significant and challenging issue to the pharmaceutical industry. The conventional method of purifying viruses by centrifugation or column-based chromatography is advantageous in that it can treat large quantities and the entire process is not complicated. However, there may be a difference in purity for each batch due to the principle of the centrifugation method, and the chromatography method using a column has the demerit in that it needs some cost. A membrane-based virus purification method can be an alternative because it can process many samples at a relatively low cost. Weigel et al. fabricated a sulfated cellulose membrane adsorber and salt-tolerant membrane adsorber through a continuous chemical reaction procedure, respectively, and applied them to the virus purification process [122]. As a result, a yield of 75% was obtained for influenza virus A/PR/8/34(H1/N1), and the levels of total protein and DNA contamination decreased to 24% and 0.5%, respectively, so that the purification performance was improved before using only a sulfated cellulose membrane adsorber. Carvalho et al. used a normal polyethersulfone (PES) membrane to perform a purification process consisting of multiple steps (using a PES membrane with different pore sizes), resulting in more than 98% of total protein and 75% of DNA being filtered [123]. Furthermore, the processing time of 30% was saved compared to the conventional virus purification process. Moreover, Carvalho et al. applied and optimized the sulfated cellulose membrane adsorber as a remover in the chromatography-based influenza virus-like particle (VLP) purification process and obtained an elimination rate of more than 89% and 80% for total protein and DNA impurities, respectively, without loss of VLP [124]. If the MMM for virus purification is further advanced and applied appropriately to various processes, the quality of virus purification or process efficiency (cost and time, etc.) can be further improved.

### 5.3. Another Application: Medical Skin Patch 

In addition to applying MMM to the above-mentioned air- and water-related research areas, there are many other research results in various fields. Among them, the field where the clear effect of MMM was shown is to use it as a skin patch for wound dressing. It is common to cover the wound with sterilized gauze to prevent further infection or secondary physical damage resulting from exposure to the atmosphere. However, normal gauze is exposed to the risk of infection because it can physically block germs on contact but cannot remove them. If gauze is replaced with MMM, which has antimicrobial properties, it not only physically protects the wound but also kills the germs or viruses on contact so that secondary infection can be more effectively prevented. The MMMs, fabricated with various functional materials such as β-chitin [128], polyurethane (PU)/N-halamine nanofiber with gelatin/rutin nanofibrous hydrogel inner layer [129], AgNO3 treated chitosan/gelatin/PU [130], chlorinated PU/polyacrylic acid nanofiber [131], N-halamine functionalized poly (ADMH-co-MMA) fiber [132], PU/keratin/Ag [133], and Fmoc-phenylalanine/Fmoc-leucine hydrogel [134], showed antimicrobial and biocompatible properties. However, there are some limitations on the selection of material because we need to manufacture MMMs using materials that have antimicrobial properties among biocompatible ones. The research on MMM that can be applied to wound dressing is actively progressing, and it is expected that production as a commercial product will be possible if the antimicrobial ability/mechanical stability/price is further improved. 

## 6. Perspectives

Currently, antiviral protective materials for both water and air filtration are the focus of research because of the COVID-19 pandemic. Most of the air and water filtration devices (masks and membranes) can only reject viruses by repelling or adsorbing them on the surface of the filtration devices. The accumulation of viral particles on filtration device surfaces can compromise the viral rejection, thus increasing the possibility of infection or permeate contamination. Similarly, public health issues due to the spread of the novel coronavirus (SARS-CoV-2) have raised some levels of concern with the current air and water treatment systems. Conventional membranes merely concentrate viruses in the feed without any deactivation activity. Air filtration using a face mask, on the other hand, can prevent airborne viral infection; however, indiscriminate handling and disposal of these masks is a major concern because of cross/secondary infection. Therefore, an effective filtration device would be that which can adequately reject viral components and at the same time deactivate viruses on contact. 

Recent advancements in the field of nanotechnology have shown the potential of this technology for antimicrobial properties. Therefore, one approach to implement this technology is to incorporate nanomaterials into the structures of filtration devices from where they can impact contact antiviral (air filtration) and contact- and release-based antiviral properties (water filtration). However, the major drawback of release-based viral killing is their short-term efficacy and non-heterogeneity of nanomaterials in the filtration device matrix. Furthermore, the fate of nanomaterials in the environment is not fully known. There is evidence to support the nontoxicity of some of the introduced nanomaterials to eukaryotic cells. Long-term and intensive studies are still required to validate this claim. In terms of filtration performance of antiviral MMMs, most of the available literature reported conclusive antiviral activity, but the exact mechanisms of action remain speculative owing to insufficient evidence. An in-depth understanding of the mechanism of antiviral nanomaterials is needed for the commercial application of antiviral MMMs. Future research is also needed in the assessment of nanomaterial-polymer compatibility to prevent the uncontrolled leaching of antiviral nanomaterials. Likewise, because one of the industrial challenges of antiviral MMM is cost, detailed studies on the reusability and longevity of antiviral MMM are urgently needed. As seen in the reported studies, the potency of antiviral nanomaterials is viral strain specific. Therefore, there is a crucial requirement to investigate the broad-spectrum antiviral properties of antiviral MMM or antiviral nanomaterials. Future research plans could focus on the combination of different antiviral nanomaterials (nanocomposites) to integrate the advantages and potency of each component of the nanocomposite. This may have the potential for a synergistic relationship, further improving the efficiency of antiviral MMM for air and water filtration. In addition, to improve the potential of antiviral MMM in the filtration field, the structural and physicochemical properties of the nanomaterials can be augmented by modifying their size and surface [56,59]. Finally, the photodynamic inhibition ability and other stimuli-responsive properties of some of the discussed nanomaterials [135] could be explored to make contact-killing more predominant than release-killing.

## 7. Conclusions

The feasibility of nanotechnology to alleviate viral infection and contamination in air and water filtration systems was discussed. The interaction of some antiviral nanomaterials and viruses was summarized with emphasis on how these nanomaterials can be incorporated into the matrix of membranes used for water and air filtration. Although some progress has been made using nanomaterials for viral control in water and air treatment, greater heights and achievement are attainable if some of the fundamental challenges discussed in this work are addressed.

## Figures and Tables

**Table 1:** Types of antiviral nanomaterials.

Nanomaterials (NMs)	Target Virus	Mode of Action	References
AgNMs	Bovine herpesvirus-1 (BoHV-1), MS2 bacteriophage, Tacaribe virus (TCRV), Hepatitis B virus (HBV),	ROS production, viral replication inhibition, direct viral inactivation, viral binding, and interaction with double-stranded DNA	[35,36,37,38]
Ag_2_S NCs	Porcine epidemic diarrhea virus (PEDV)	Blockage of viral DNA and budding	[39]
AuNMs	Measles virus (MeV), Respiratory syncytial virus (RSV)	Direct virus blocking and viral replication inhibition	[40,41]
CuNMs	Herpes simplex virus−1 (HSV−1), hepatitis C virus (HCV)	Viral proteins and genome oxidation (ROS) and degradation, viral protein denaturation	[42,43]
ZnO NMs	Human influenza A virus (H1N1), HSV−1	Polymerase function inhibition, contact killing	[44,45,46]
TiO_2_ NMs	Newcastle disease virus (NDV)	Viral lipid envelope destruction	[47,48]
Carbon-based NMs (fullerene, SWCNTs, MWCNTs, graphene, graphene oxide)	Human immunodeficiency virus (HIV−1), HSV−1,2, coxsackievirus (Cox B3), cytomegalovirus, grass carp reovirus (GCRV)	Viral maturation and replication inhibition interact with viral enzyme, RNA polymerase inhibition, viral membrane destruction	[49,50,51,52,53]
Silica-based NMs	RSV, HIV	Viral inactivation, disruption of DNA replication	[54,55]
SnO_2_ NMs	HSV−1	Inhibition of cell entry and cell-to-cell spread	[56]

**Table 2:** Application of antiviral nanomaterials-incorporated MMMs.

Application	Material	Membrane Structure	Ref.
Air purification	ZnO	Nanocomposite	[113]
Air purification	ZnO/Ag	SBS nanofibers	[114]
Air purification	CTAB@MGO	Nanocomposite	[115]
Air purification	Cellulose/silica BNCs	Nanocomposite	[116]
Air purification	PANI nanorods	Nanocomposite	[117]
Air purification	Herbal extract	HEI nanofiber	[118]
Sanitation in ultrafiltration	Ag@TiO_2_/crumpled GO	Stacked composite	[119]
Sanitation in water treatment	TiO_2_-decorated Nylon nanofiber	Nanofiber membrane	[120]
Sanitation in medical tool	Peptide-functionalized sodium hyaluronate and chitosan	Layered coating	[121]
Virus purification	Sulfated cellulose	Film on support	[122]
Virus purification	Polyethersulfone	Thin-film composite	[123]
Virus-like particle purification	Sulfated cellulose	Film on support	[124]

## Data Availability

Not applicable.
